# Structured sparse multiset canonical correlation analysis of simultaneous fNIRS and EEG provides new insights into the human action-observation network

**DOI:** 10.1038/s41598-022-10942-1

**Published:** 2022-04-27

**Authors:** Hadis Dashtestani, Helga O. Miguel, Emma E. Condy, Selin Zeytinoglu, John B. Millerhagen, Ranjan Debnath, Elizabeth Smith, Tulay Adali, Nathan A. Fox, Amir H. Gandjbakhche

**Affiliations:** 1 Eunice Kennedy Shriver National Institute of Child Health and Human Development (NICHD), National Institutes of Health, Bethesda, MD USA; 2 Department of Human Development and Quantitative Methodology, University of Maryland, College Park, MD USA; 3 Leibniz Institute for Neurobiology, Magdeburg, Germany; 4 Behavioral Medicine and Clinical Psychology Department, Cincinnati Children’s Hospital Medical Center, Cincinnati, OH USA; 5 Department of Computer Science and Electrical Engineering, University of Maryland Baltimore County, Baltimore, MD USA

**Keywords:** Perception, Data integration

## Abstract

The action observation network (AON) is a network of brain regions involved in the execution and observation of a given action. The AON has been investigated in humans using mostly electroencephalogram (EEG) and functional magnetic resonance imaging (fMRI), but shared neural correlates of action observation and action execution are still unclear due to lack of ecologically valid neuroimaging measures. In this study, we used concurrent EEG and functional Near Infrared Spectroscopy (fNIRS) to examine the AON during a live-action observation and execution paradigm. We developed structured sparse multiset canonical correlation analysis (ssmCCA) to perform EEG-fNIRS data fusion. MCCA is a generalization of CCA to more than two sets of variables and is commonly used in medical multimodal data fusion. However, mCCA suffers from multi-collinearity, high dimensionality, unimodal feature selection, and loss of spatial information in interpreting the results. A limited number of participants (small sample size) is another problem in mCCA, which leads to overfitted models. Here, we adopted graph-guided (structured) fused least absolute shrinkage and selection operator (LASSO) penalty to mCCA to conduct feature selection, incorporating structural information amongst the variables (i.e., brain regions). Benefitting from concurrent recordings of brain hemodynamic and electrophysiological responses, the proposed ssmCCA finds linear transforms of each modality such that the correlation between their projections is maximized. Our analysis of 21 right-handed participants indicated that the left inferior parietal region was active during both action execution and action observation. Our findings provide new insights into the neural correlates of AON which are more fine-tuned than the results from each individual EEG or fNIRS analysis and validate the use of ssmCCA to fuse EEG and fNIRS datasets.

## Introduction

Recent developments in non-invasive neuroimaging techniques have allowed for multi-modal measurement of brain activation, leading to more comprehensive understanding of the neural processes underlying cognition. One multi-modal approach is the combination of electrophysiological data derived from electroencephalography (EEG) with hemodynamic response data derived from functional near-infrared spectroscopy (fNIRS)^1,2^. EEG records electrical signal changes from neuronal firing through electrodes attached to the scalp. FNIRS is an optical neuroimaging technique that uses near-infrared light (670–820 nm wavelength) to measure the hemodynamic response in the cerebral cortex, providing an indirect measure (e.g., blood oxygenation) of neuronal activation. Although other methods can also measure the hemodynamic response, such as functional magnetic resonance imaging (fMRI), fNIRS is non-invasive, cost effective, easier to apply, and less susceptible to motion artifact, making it more amenable to studies related to motor activity^3,4^. EEG and fNIRS are a compelling combination of modalities due to their complementary features. EEG captures electrical activity with temporal resolution on the order of milliseconds, but lacks spatial resolution. Conversely, fNIRS has improved spatial resolution^5–7^ and greater tolerance to motion artifact than EEG, allowing the identification of specific regions of interest and greater flexibility in the behavioral paradigms employed. By combining these modalities, better understanding of neural activation can be achieved by leveraging the temporal resolution of EEG with the improved spatial resolution and methodological flexibility of fNIRS. Using this combined approach, studies of brain activation associated with live action or motor-based tasks may yield more specific, robust findings.

Concurrent recording of EEG and fNIRS could be particularly beneficial in understanding the human action-observation network (AON). The AON is a network of brain regions linking the actions of the self (action execution) to the actions of others (action observation)^8^. It has been proposed that the AON is associated with sophisticated social and learned behaviors that emerge in typically developing infants, such as complex imitation and shared emotion^9^. The AON has been widely studied in humans using EEG, namely through measurement of mu (μ) desynchronization^10^. It is suggested that μ desynchronization occurs both when someone performs an action and when they observe someone perform the same action, and thus has been the predominant measure of the AON in the human brain^11^. However, whether μ desynchronization truly reflects a mirroring system in the human brain remains controversial^12,13^. One reason for this controversy is the lack of spatial specificity in the measured neural response provided by EEG. fMRI studies have offered a more precise location of AON related brain activity^14,15^ and have identified a key set of regions, including the ventral premotor cortex (PMv), inferior frontal gyrus (IFG), and inferior parietal lobe (IPL)^16,17^. However, fMRI studies are limited due to their high sensitivity to motion artifact, which is problematic in AON experimental protocols that involve the execution of a motor behavior. Most fMRI studies do not include an execution condition^15^ or, if they do, the actions employed might not be ecologically valid (e.g., bite bars, ball squeezing)^18,19^. While the confines of fMRI are not ideal for adequate experimental designs of AON experiments, there is potential for fNIRS to fill this gap as it has increased spatial resolution compared to EEG and allows participants to move during data collection unlike fMRI^3^.

Combining data from multiple neuroimaging techniques could be useful in understanding brain mechanisms related to the AON^20^, but the analysis of multimodal information is inherently challenging. Canonical Correlation Analysis (CCA) is a classic way to evaluate the multivariate associations between two types of high dimensional data using canonical vectors or matrices (e.g., different neuroimaging modalities)^21^. However, CCA is designed for two datasets. Multiset CCA (mCCA) extends CCA to more than two datasets^22^. While CCA maximizes the correlation between two canonical variates, mCCA optimizes an objective function of the correlation matrix of canonical variates from multiple random vectors such that the correlation between canonical variates is maximized. This method has been applied to fMRI datasets on multiple participants^23–27^, multi-subject EEG datasets^28,29^, fMRI and EEG multi-subject datasets^30^, EEG and magnetoencephalography (MEG) multi-subject datasets^31^, and fMRI, structural MRI and EEG datasets^32–34^. There have also been previous studies using simultaneously recorded EEG and fNIRS. They have used a variety of data fusion approaches, including joint independent component analysis^35^, CCA^5,6^ or temporally encoded CCA^36^. However, despite the strengths and applicability of CCA in such studies, it performs poorly when the number of features (e.g., brain regions) exceeds the number of observations (e.g., participants), which is often true in neuroimaging data analysis. This makes overfitting and limited generalizability prominent obstacles to overcome when using this approach.

While CCA does not perform feature selection, sparse CCA (sCCA) contains a built-in procedure to address this concern. SCCA extends on CCA by using a regularization technique to identify a sparse set of canonical vectors (loading/projection vectors) for both sets of features. Although sCCA offers a simpler interpretation by creating sparse projection vectors with higher correlations, it does not consider the spatial correlation or structural relationship between input features (e.g., brain regions). This can limit usefulness of sCCA in high-dimensional data fusion looking to localize the neural response. Since neural activity in adjacent regions is likely more similar than neural activity in non-adjacent regions, correlating them so that their canonical coefficients have similar magnitudes can address this issue. Witten et al.^37^ introduced structured sCCA (ssCCA), which imposes a fused least absolute shrinkage and selection operator (LASSO) penalty^38^ that tends to group neighboring features to recover their spatial structure.

The present study uses a multiset version of sparse structured CCA (ssmCCA) to examine brain signals derived from concurrent EEG and fNIRS during an action execution-observation task. Specifically, ssmCCA is used to decode and fuse electrical and hemodynamic responses associated with neural activation to expand our understanding of brain activation during AON (execution and observation) conditions. Using a multimodal neuroimaging approach combining EEG and fNIRS to investigate the AON we aim to identify more specific regions of interest in the brain and further advance our understanding of social neuroscience. We propose that if EEG and fNIRS each contain greater temporal or spatial information respectively, then a multimodal imaging approach using fusion analysis will yield more specific brain activation patterns than either EEG or fNIRS when analyzed alone. This will be assessed by comparing the activation patterns yielded from the unimodal analyses of fNIRS and EEG to the multimodal approach using ssmCCA. This paper is organized as follows: first we introduce the mathematical approach behind ssmCCA, then the details of the experiment and data preprocessing for both fNIRS and EEG datasets are presented. The data fusion procedure is then applied and findings from this approach compared to unimodal EEG and fNIRS analyses are presented, followed by a discussion interpretting our results and relating them to the previous literature.

## Method

### Data fusion approach

#### Structured sparse CCA (ssCCA)

CCA is a standard method to explore the relationship between two sets of multi-dimensional variables. When the number of samples (i.e., participants) are smaller than the number of features ($n \ll p$ and $n \ll q$), overfitting becomes an inevitable problem. Usually in this situation, only fractions of features in each set are necessary for characterizing the two-set correlation. To mitigate the overfitting and lack of generalization problem, a sparsity constraint has been added to the traditional CCA problem^37,39^. To make both canonical variates sparse, either an *l1*-norm (LASSO) term^40,41^ or a combination of *l1*-norm and *l2*-norm (fused LASSO)^38,42^ are added to the traditional CCA model. Recently, there have been a number of structured sparse CCA (ssCCA) approaches proposed using graph/network-guided fused LASSO penalties^43–46^. In this paper, we take advantage of the model suggested by He^47^ where a network is represented by undirected weighted graph, ${\mathcal{G}}$. GraphNet, a more general form of a traditional elastic net regularizer^43–46^, can be written as:

1 $${\left\| u \right\|}_{{\mathcal{G}}} = { }\lambda_{1} { }u^{T} M{ }u + {\gamma }_{1} { }{\left\| u \right\|}_{1}$$

where $M$ is a matrix, and ($\lambda_{1} ,\gamma_{1}$) are regularizing parameters. The vertices in ${\mathcal{G}}$ correspond to features (e.g., brain regions, optodes) and each edge, $l_{ij}$, indicates if there is a link between optode $i$ and $j$ in ${\mathcal{G}}$; all the weights of $l_{ij}$ in the network depend on their adjacency conditions (e.g., high or low correlation). We have an elastic net when $M = I$ in a GraphNet. Generally, $M = L$, where $L$ is the Laplacian matrix of a graph. Let $A$ be a sample correlation matrix, called the adjacency matrix, in which the higher pairwise correlation between two features corresponds to a larger weight. We identify $p$ as features/brain regions in the dataset and a diagonal matrix, $D$, with the following diagonal entries: ($D = { }diag(d_{1} ,{ }d_{2} , \ldots ,d_{p}$), where $D\left( {i,i} \right) = { }\mathop \sum \limits_{j = 1}^{p} A\left( {i,j} \right)$. The Laplacian matrix, $L$, is defined as $L = D - W$. In the case of $M = L$, it has been shown that:

$$u^{T} L{ }u = { }\mathop \sum \limits_{1 \le i \le k \le p} a_{ik} \left( {u_{i} - { }u_{k} } \right)^{2}$$

where $w_{ik}$ depends on pairwise correlation of $X$ and $Y$ respectively^47^. This cost function also indicates that the adjacent regions linked in initial structure are expected to have similar weights^48,49^. To this end, the ssCCA would be formulated as:

2 $$\mathop {\max }\limits_{{w_{1} , w_{2} }} w_{1}^{T} X^{T} Y w_{2 }$$

subject to:

$$\left\{ {\begin{array}{*{20}l} {\left\| {w_{1} } \right\| _{2}^{2} \le 1,} \hfill & {\left\| {w_{2} } \right\| _{2}^{2} \le 1,} \hfill \\ {\left\| {w_{1} } \right\| _{1} \le c_{1} ,} \hfill & {\left\| {w_{2} } \right\| _{1} \le c_{2} ,} \hfill \\ {w_{1}^{T} L_{w1} w_{1} \le c_{3} ,} \hfill & {w_{2}^{T} L_{w2} w_{2} \le c_{4} } \hfill \\ {w_{1} \bot w_{2} } \hfill & {} \hfill \\ \end{array} } \right.$$

where C = ($c_{1} , c_{2} , c_{3} ,c_{4}$) > 0 are regularization parameters. Here, $c_{3}$ and $c_{4}$ are used to regularize the cost function controlling for spatial structure, and $L_{w1}$ and $L_{w1}$ are Laplacian matrices of modality 1 and 2 associated with $w_{1}$ and $w_{2} ,$ respectively. Witten et al.^37^, Chen et al.^43^ reformulated the constraints on $w_{1}$ and $w_{2}$ in Lagrangian form:

3 $$\mathop {\max }\limits_{{w_{1} , w_{2} }} w_{1}^{T} X^{T} Y w_{2} - \lambda_{1} \left\| {w_{1} } \right\|_{1} - \lambda_{2} \left\| {w_{2} } \right\|_{1} - \frac{1}{2} w_{1}^{T} \left( { I + \alpha_{1} L_{w1} } \right) w_{1 } - \frac{1}{2} w_{2}^{T} \left( { I + \alpha_{2} L_{w2} } \right) w_{2}$$

where the regularization parameters $\lambda_{1} , \lambda_{2} , \alpha_{1}$ and $\alpha_{2}$ correspond to $c_{1} , c_{2} , c_{3}$ and $c_{4}$, in (3) respectively.

#### Structured sparse multiset CCA (ssmCCA)

Thus far, the ssCCA we have formulated does not consider more than two datasets. However, traditional CCA can be extended to more than two variables in different ways^22^. MCCA is the generalization of the CCA model where an objective (cost) function corresponding to the correlations between canonical vector pairs should be optimized such that the overall correlation between them is maximized: $\left[ {w_{1}^{\left[ 1 \right]} ,w_{2}^{\left[ 1 \right]} \cdots w_{M}^{\left[ 1 \right]} } \right]$ = $\mathop {{\text{max}}}\limits_{w}$($|\rho_{k,l}^{\left[ n \right]} |)$. The subscript indicates the dataset, and the superscript indicates the observation in the dataset (e.g., $\rho_{k,l}^{\left[ n \right]}$ is the correlation between the $n$ th canonical variates from $k$ th and $l$ th datasets).

In order to solve mCCA Kettenring^22^ introduced five objective functions (e.g., ${\mathcal{F}}\left( { x} \right) = x$ indicates the sum of correlations (SUMCOR) cost function while ${\mathcal{F}}\left( { x} \right) = x^{2}$ corresponds to the sum of squares correlations (SSQCOR) cost function):

4 $$\mathop {\max }\limits_{w} \mathop \sum \limits_{k,l = 1}^{M} {\mathcal{F}}_{k,l} \left( { \cdot } \right) \quad k \ne l$$

where function ${\mathcal{F}}\left( \cdot \right)$ is the cost function. Here, we chose the SUMCOR cost function to estimate canonical variates. The procedure can be summarized in the steps bellow.

Step 1

5 $$\left[ {w_{1}^{\left[ 1 \right]} ,w_{2}^{\left[ 1 \right]} \ldots w_{M}^{\left[ 1 \right]} } \right] = \mathop {\max }\limits_{w} \left\{ {\mathop \sum \limits_{k,l = 1}^{M} |\rho_{k,l}^{\left[ 1 \right]} | } \right\} = \mathop {\max }\limits_{w} \mathop \sum \limits_{k,l = 1}^{M} {\mathcal{F}}\left( { w_{k}^{\left[ 1 \right]T} X_{k}^{T} X_{l} w_{l}^{\left[ 1 \right]} } \right)$$

Step 2

6 $$\left[ {w_{1}^{\left[ n \right]} ,w_{2}^{\left[ n \right]} \ldots w_{M}^{\left[ n \right]} } \right] = \mathop {{\text{max}}}\limits_{w} \left( { \mathop \sum \limits_{k,l = 1}^{M} |\rho_{k,l}^{\left[ n \right]} | } \right)$$

subject to:

$$\left\{ {\begin{array}{*{20}l} {\left( a \right)\;\left\| {w_{k}^{\left[ n \right]} } \right\|_{2}^{2} \user2{ } \le 1, \quad \quad \left\| {w_{l}^{\left[ n \right]} } \right\|_{2}^{2} \user2{ } \le 1} \hfill & {m = 1, \ldots ,M} \hfill \\ {\left( b \right)\left\| {w_{k}^{\left[ n \right]} } \right\|_{2}^{2} \user2{ } \le c1_{k}^{\left[ n \right]} , \quad \quad \left\| {w_{l}^{\left[ n \right]} } \right\|_{2}^{2} \user2{ } \le c1_{l}^{\left[ n \right]} ,} \hfill & {n = 1, \ldots ,N} \hfill \\ {\left( c \right) w_{k}^{\left[ n \right]T} L_{{w_{k}^{\left[ i \right]} }}^{\left[ n \right]} w_{k}^{\left[ n \right]} \le c2_{k}^{\left[ n \right]} w_{l}^{\left[ n \right]T} L_{{w_{l}^{\left[ i \right]} }}^{\left[ n \right]} w_{l}^{\left[ n \right]} \le c2_{l}^{\left[ n \right]} ,} \hfill & {1 \le k,l \le M} \hfill \\ {\left( d \right) w_{m}^{\left[ n \right]} \bot \left\{ {w_{m}^{\left[ 1 \right]} , \ldots ,w_{m}^{\left[ N \right]} } \right\}} \hfill & {k \ne l} \hfill \\ \end{array} } \right.$$

where $d \le \min \left( {{\text{rank}}\left( {X_{m} } \right)} \right)$. The matrices $L_{{w_{m}^{\left[ n \right]} }}^{\left[ n \right]}$ are the semi-positive definitive Laplacian matrices of $M$ datasets, and $c1_{m}^{\left[ n \right]}$ and $c2_{m}^{\left[ n \right]}$ are the penalty terms for the $n$ th observation throughout all the $M$ datasets.

Step (1) can be solved using a partial derivative function of the SUMCOR objective function with respect to each $w_{m}^{\left[ 1 \right]}$ and equating it to zero to find the optimizing point. Since the SUMCOR objective function is a linear function of each $w_{m}^{\left[ 1 \right]}$, the partial derivative is a constant, and therefore the closed form solution exists. The iterative algorithm starts from randomly initializing canonical variates and each $w_{k}^{\left[ 1 \right]}$ vector is updated subsequently to guarantee cost function optimization. All the $w_{k}^{\left[ 1 \right]}$ vectors are updated after the one step procedure. The procedure continues until convergence criteria are met and the $w_{k}^{\left[ 1 \right]}$ vectors are considered as the optimal solution.

At step (2), the SUMCOR problem in (8) can then be reformulated in Lagrangian form:

7 $$\begin{aligned} & \mathop {\max }\limits_{{w_{k,l}^{\left[ n \right]} }} w_{k}^{\left[ n \right]T} X_{k}^{T} X_{l} w_{l}^{\left[ n \right]} - \lambda_{1k}^{\left[ n \right]} \left\| {w_{k}^{\left[ n \right]} } \right\|_{1} - \lambda_{2l}^{\left[ n \right]} \left\| {w_{l}^{\left[ n \right]} } \right\|_{1} \\ & \quad \quad \quad - \frac{1}{2} w_{k}^{\left[ n \right]T} \left( { I + \alpha_{1k}^{\left[ n \right]} L_{{w_{l}^{\left[ n \right]} }} } \right)w_{1} \\ & \quad \quad \quad - \frac{1}{2} w_{l}^{\left[ n \right]T} \left( { I + \alpha_{2k}^{\left[ n \right]} L_{{w_{k}^{\left[ n \right]} }} } \right) w_{2} \\ \end{aligned}$$

where 1 $\le n \le N$, $1 \le k,l \le M$, $k \ne l.$ As a reminder, $n$ represents the $n$ th observation in $k$ th and $l$ th datasets, and the regularization parameters $\lambda_{1k}^{\left[ n \right]} , \lambda_{2k}^{\left[ n \right]} , \alpha_{1k}^{\left[ n \right]}$ and $\alpha_{2k}^{\left[ n \right]}$ correspond to $c1_{k}^{\left[ n \right]}$, $c2_{k}^{\left[ n \right]}$ , $c1_{l}^{\left[ n \right]}$ and $c2_{l}^{\left[ n \right]}$ in (8) respectively.

#### Parameter optimization

The parameters in (9) that should be optimized are ($\lambda_{1k}^{\left[ n \right]} , \lambda_{2k}^{\left[ n \right]} , \alpha_{1k}^{\left[ n \right]}$, $\alpha_{2k}^{\left[ n \right]}$). We applied the leave-one-out cross validation technique in which we estimated the model parameters (canonical variates) for $n$ − 1 samples (participants) and calculated the errors on our one-left-out sample. We adopted the two steps cross-validation technique^50^. First, we found optimal values of $\alpha_{i}$ when $\lambda_{i}$ was set to zero and, second, we used these optimal $\alpha_{i}$ values to estimate optimal values of $\lambda_{i}$. To address the overfitting problem and improve model generalization, Waaijenborg et al.^51^ suggested that the test sample correlation should be approximately equal to the training sample correlation. In other words, the absolute difference between the estimated canonical correlations of the training and test samples are minimized.

### Participants

Data were collected at two sites: the National Institute of Health (NIH) and University of Maryland (UMD). At NIH, participants were recruited from the healthy volunteer database at the National Institutes of Health. At UMD, participants were recruited through the program for Research for Extra Credit supported by the Department of Psychology. All experiments and methods were performed in accordance with guidelines provided in the study protocol (number: 18-CH-0001), which was approved by the Office Of Research Support and Compliance (ORSC) at NIH. In addition, all participants signed an informed consent approved by each site’s Institutional Review Board (IRB) prior to the start of the experiment.

Forty healthy right-handed volunteers participated in the experiment at NIH the site; however, data from 27 participants had to be discarded as a result of technical malfunctions which caused either incomplete recording or poor signal quality in one or both modalities. The final sample consisted of seven females and six males. Twenty healthy volunteers participated at UMD. Data from eight participants was considered for further analysis (3 female and 5 male, mean age, 24.62 years). Between both sites, the final sample used for the data fusion algorithm consisted of 21 participants (22–29 years of age; mean age, 24.9 years).

### Experimental design

Our experiment was adapted from a paradigm used in the EEG mirror neuron literature with infant populations^52^. The paradigm consisted of 15 trials of action observation and 15 trials of action execution. Each trial was followed by a 20 s recovery period during which the participant passively viewed a moving pendulum. For further information on the paradigm we used please see Miguel et al.^53^.

### Data acquisition

EEG and fNIRS data were recorded simultaneously. EEG data were collected using the Geodesic EEG System 400 (Magstim EGI, Eugene, OR) with 128 electrodes at a sampling rate of 256 Hz. Participants’ heads were fit with elastic EGI Geodesic Sensor Nets based on their head size. We measured head circumference, nasion-to-inion, and preauricular point distances to ensure proper placement of the EEG cap. The vertex (Cz) electrode was used as the reference. Data were exported to a MATLAB (Mathworks, Natick, MA) compatible format using Net Station software for offline processing with the EEGLab (v13.4.4b) toolbox^54^.

fNIRS data were collected using the Hitachi ETG-4100 system equipped with 10 infrared sources and 8 detectors placed over the somatosensory and parietal regions as in our previous fNIRS study investigating the AON^53^. A total of 24 channels (12 per hemisphere) measured changes in oxygenated hemoglobin (HbO) and deoxyhemoglobin (HbR) concentration.

**Figure 1:**
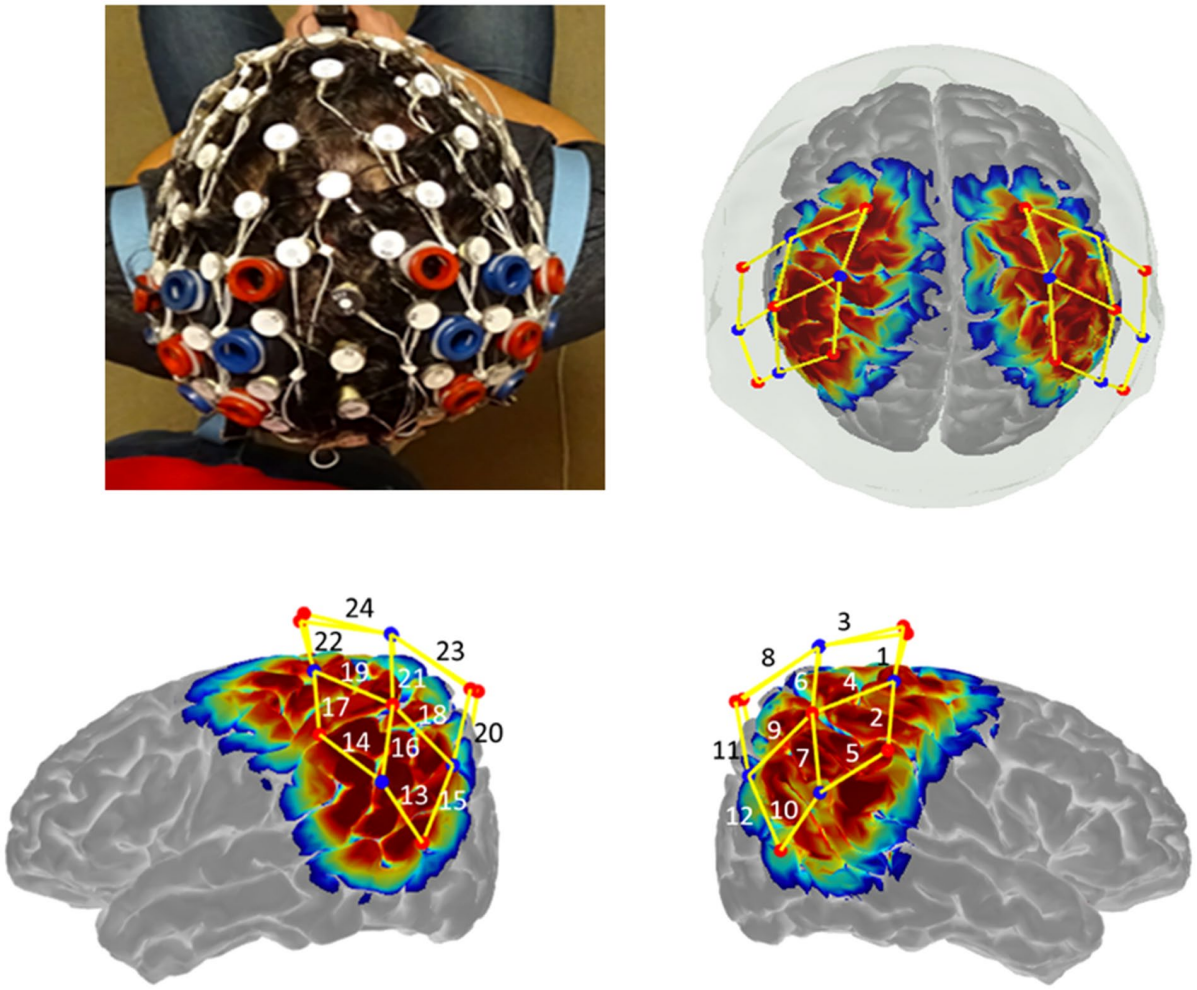
fNIRS probe design. The picture on the top left shows how the fNIRS probe was embedded within an elastic, 128-electrode electroencephalogram (EEG) cap^53^. The top right, bottom left, and bottom right pictures depict the sensitivity profile for the fNIRS probe geometry generated in AtlasViewer software. The color scale indicates the relative sensitivity in log 10 units from − 1 (blue) to 1 (red). Dots represent source and detector pairs; yellow lines indicate fNIRS measurement channels. Six ROIs were derived from the 12 fNIRS channels located in each hemisphere: pre-central region, post-central region, superior parietal lobule, inferior parietal lobule, supra-marginal gyrus and angular gyrus.

After collecting experimental EEG/fNIRS data, we recorded the positions of sources and detectors on the head in reference to the nasion, inion, and preauricular landmarks using a 3D-magnetic space digitizer (Fastrak-Polhemus). This accounted for additional variations in cap placement and verified which channels covered each brain region. Figure 1 shows how fNIRS optodes were secured within the elastic of the EEG cap using custom-designed silicone fixtures, as well as the sensitivity profile of the fNIRS probe across the cortex.

### Preprocessing

#### EEG data preprocessing

EEG data were pre-processed using EEGLab software using the method proposed by Debnath and colleagues^55^. EEG channels on the boundary of the electrode net (24 channels) were excluded from analyses because they were contaminated by eye, face, and head movements (17, 38, 43, 44, 48, 49, 113, 114, 119, 120, 121, 125, 126, 127, 128, 56, 63, 68, 73, 81, 88, 94, 99, 107). Then, high-pass and low-pass filters with respective cut off frequencies of 0.3 and 49 Hz were applied to the continuous data. Using the EEGLAB plugin FASTER^56^, artifactual channels were identified and subsequently removed. In order to remove eye blinks, respiratory, and muscle movement noise in the data, we applied extended infomax independent component analysis (ICA). During preprocessing we used the interpolation option to estimate electrical activity of the noisy electrodes mentioned above that had been removed prior to the ICA. This is a popular and well-established technique that has been mentioned in the EEGLAB tutorial. Using the ADJUST plugin^57^ to EEGLAB, artifactual independent components (ICs) were removed and the remaining data were epoched into − 1.5 s to 1.5 s intervals relative to the “Start Action” (SA) marker for each trial. Therefore, we extracted a total of 30 segments encompassing the 15 trials of each condition (observation and execution). These preprocessed data were used to conduct unimodal EEG analyses to assess AON activity as detected by EEG alone and were also carried forward into the EEG-fNIRS fusion analysis.

#### fNIRS data preprocessing

The fNIRS signal was processed using HOMER2 (MGH—Martinos Center for Biomedical Imaging, Boston, MA, USA), a MATLAB software package (The MathWorks, Inc., Natick, MA, USA). Only valid trials as identified through behavioral coding were retained in HOMER2 for data processing. Using the optical density data, we used Principal Component Analysis (PCA) set at 0.9 for movement artifact removal^58^. Data were low-pass filtered at 0.5 Hz to remove physiological influence from the signal and were then used to calculate the change in concentration of the hemoglobin chromophores using to the modified Beer-Lambert Law^59^. Traces were then segmented into 25-s epochs around the trigger stimulus for each trial (start action; SA), with each epoch starting − 5 s prior to each stimulus (0 s). Baseline correction corresponded to the mean HbO/HbR values from − 5 to 0 s. The hemodynamic response function was then generated at each channel for each condition by participant by averaging all of a participant’s response curves from all trials within a condition into a single hemodynamic curve for each channel. Due to a greater signal to noise ratio we only used the HbO signal for remaining analyses, similar to previous work in fNIRS^60^.

#### Mapping fNIRS electrodes via Atlas Viewer

We determined the anatomical regions covered by each fNIRS channel within each participant using the optode coordinates taken from the Polhemus digitizer. These coordinates were then entered into AtlasViewer^61^ to scale the Colin29 brain atlas to each participant’s head. AtlasViewer generated the MNI coordinates of each channel and the corresponding region of interest (ROI) for each channel was identified. Due to differences in head size, channels were not consistently positioned over the same brain region for all participants. Hence, the analyses were conducted using an ROI approach. The ROIs indicated were: postcentral, precentral, supra-marginal, inferior parietal and angular, located in both left and right hemisphere. Using the preprocessed fNIRS data and the ROI data, unimodal fNIRS analyses were conducted to assess AON activity as detected by fNIRS alone and were also carried forward into the EEG-fNIRS fusion analysis.

### EEG-fNIRS data fusion

First, we convolved the mean power in the $\mu$ frequency band (8–13 Hz in adults) for each EEG channel with the hemodynamic response function (HRF) using a gamma distribution^62^. AON activation in EEG is characterized by decreased power in the $\mu$ frequency band, whereas in fNIRS, AON activation is indexed by an increase in HbO over specific brain regions, specifically bilateral superior parietal lobule, bilateral inferior parietal lobule, right supra-marginal region and right angular gyrus^53^. To account for the power decrease in EEG, we used the inverse value of the power in the $\mu$ frequency band for EEG prior to applying the HRF convolution. We also used a 1000 Hz sampling rate to resample the EEG data. Since SA markers were considered the set point (0) over a 3-s epoch from − 1.5 s to 1.5 s, a total of 3000 datapoints were extracted. This resulted in the EEG matrix $E \in { }R^{{samples{ } \times { }channels}} { }\left( {R^{{3000 \times { }128}} } \right)$.

**Figure 2:**
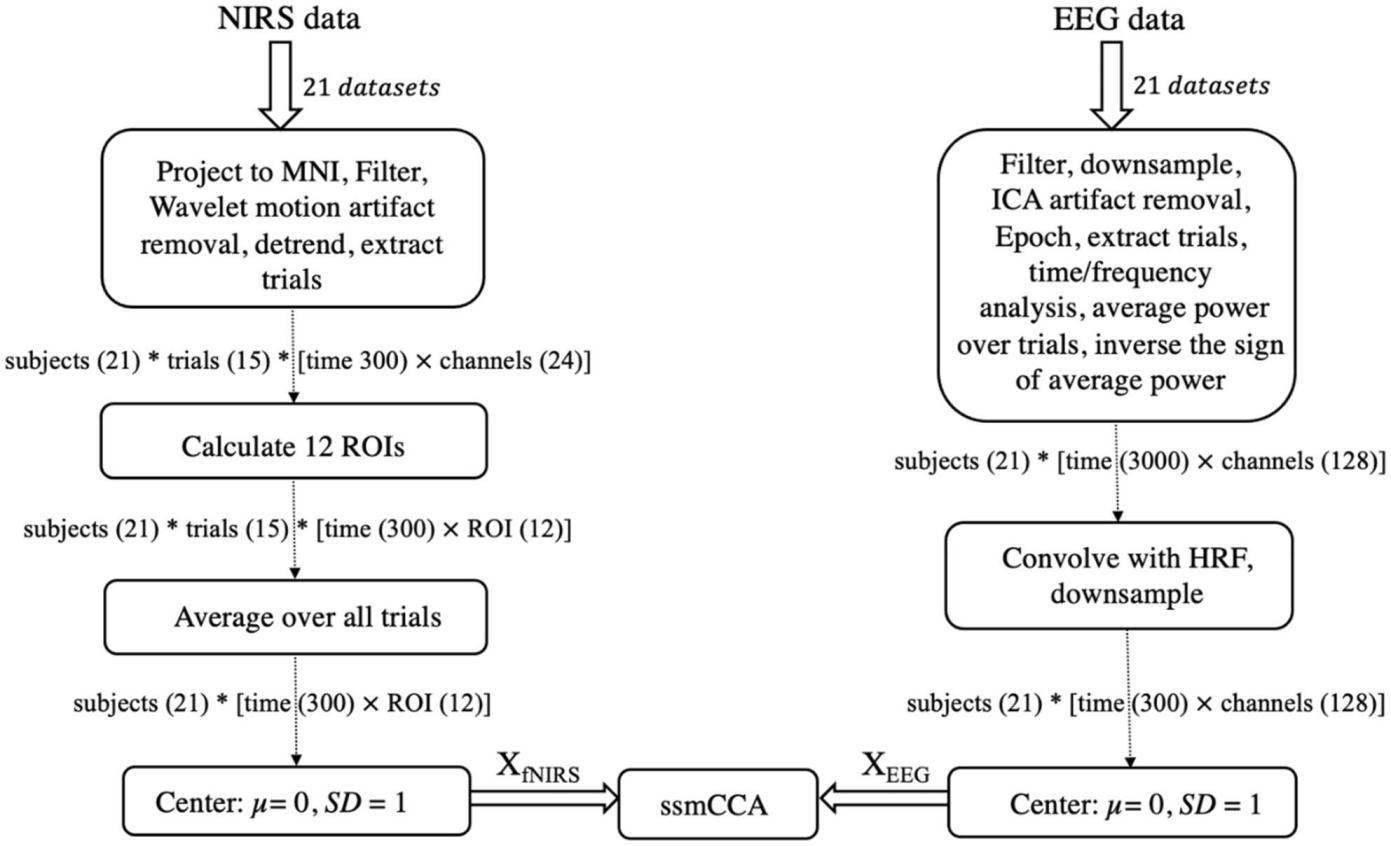
Preprocessing workflow for EEG and fNIRS datasets. After preprocessing, 42 datasets in total entered the ssmCCA algorithm.

fNIRS data were averaged over all the 30-s trials, which consisted of − 5 s before the stimulus and 25 s after at each channel. The SA marker was considered as the zero point in time in both the fNIRS and EEG datasets. The fNIRS signal was sampled at a rate of 10 Hz, resulting in an fNIRS matrix for each participant of $N \in { }R^{{samples{ } \times { }channels}} { }\left( {R^{{300 \times { }24}} } \right)$. After projecting the fNIRS dataset to MNI space for each subject, we used the 12 regions of interest identified in AtlasViewer (see preprocessing, Sect. 2.4.3). Hence, the final fNIRS data matrix was $\in { }R^{{samples{ } \times { }ROIs}} { }\left( {R^{{300 \times { }12}} } \right)$. Since CCA requires the same number of data samples (though a different number of features is still possible), we downsampled the EEG datasets. The final EEG and fNIRS datasets had the following dimensions: $E$ $\in { }R^{{300 \times { }128}}$, $N$ $\in { }R^{{300 \times { }12}}$, respectively. Figure 2 illustrates our preprocessing pipeline.

We randomly divided 21 datasets (overall 42 since each participant had two sets of data: one EEG and one fNIRS dataset) into two subsets: a training set and test set. The optimal parameters were obtained from the training dataset by threefold cross validation. Then, we used the estimated parameters on the test sets to predict the correlation between the datasets. The main reason we chose threefold cross validation over the leave-one-out technique is the lower variance provided by this method. In the case of leave-one-out, where 90% of data are used for training and 10% used for testing, the test set is very small, so there is high variation in the performance estimate across different samples of data and across the different partitions of the same data forming the training and test sets. threefold validation reduces this variance by averaging over 3 different partitions, so the performance estimate is less sensitive to the partitioning of the data. We also repeated threefold cross-validation, where the cross-validation is performed using different partitioning of the data to form 3 subsets, and then took its average as well.

## Results

### Unimodal fNIRS

**Figure 3:**
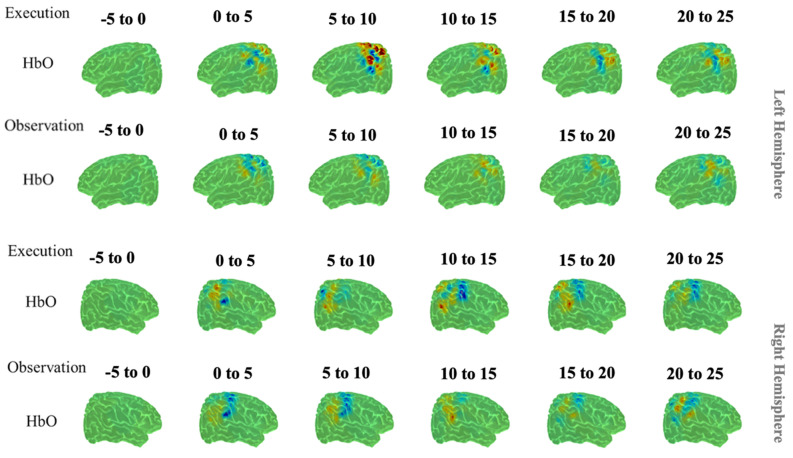
Unimodal fNIRS results. HbO reconstruction maps for Execution and Observation in the Left and Right Hemisphere from − 5 to 25 s. Overall, our results showed the parietal regions, including bilateral superior parietal lobule, bilateral inferior parietal lobule, right supra-marginal region and right angular gyrus are candidate regions of the human AON^53^.

FNIRS findings suggested that bilateral superior parietal lobule (SPL), bilateral inferior parietal lobule (IPL), right supra-marginal region (SMG) and right angular gyrus (AG) are candidate regions of the human AON, as previously reported in Miguel et al.^53^. Figure 3 represents a summary of our findings.

### Unimodal EEG

**Figure 4:**
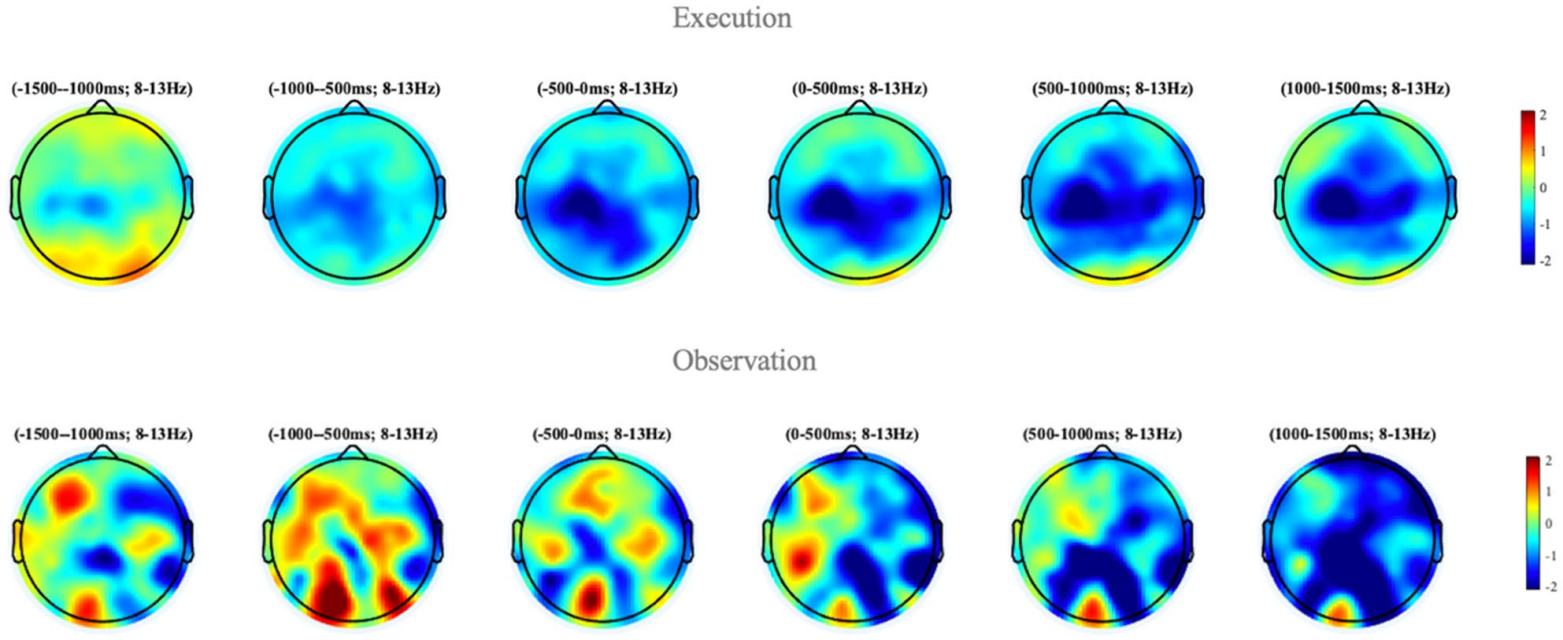
Unimodal EEG results. Our EEG results show strong μ desynchronization during execution and observation conditions. Here power synchronization is indicated by warmer colors on the colorbar, while desynchronization is shown using cooler colors. Thus, μ desynchronization can be seen in blue for both action execution and action observation. However, the source of μ desynchronization is unspecified across the cortex due to poor spatial resolution of EEG.

Results from EEG unimodal analysis indicated bilateral μ desynchronization in central and parietal regions for execution, whereas observation resulted in bilateral μ desynchronization in the parietal region (unimodal EEG findings are in the process of publication). Figure 4 provides a topographic view of the μ desynchronization from the EEG data.

### EEG-fNIRS fusion

**Figure 5:**
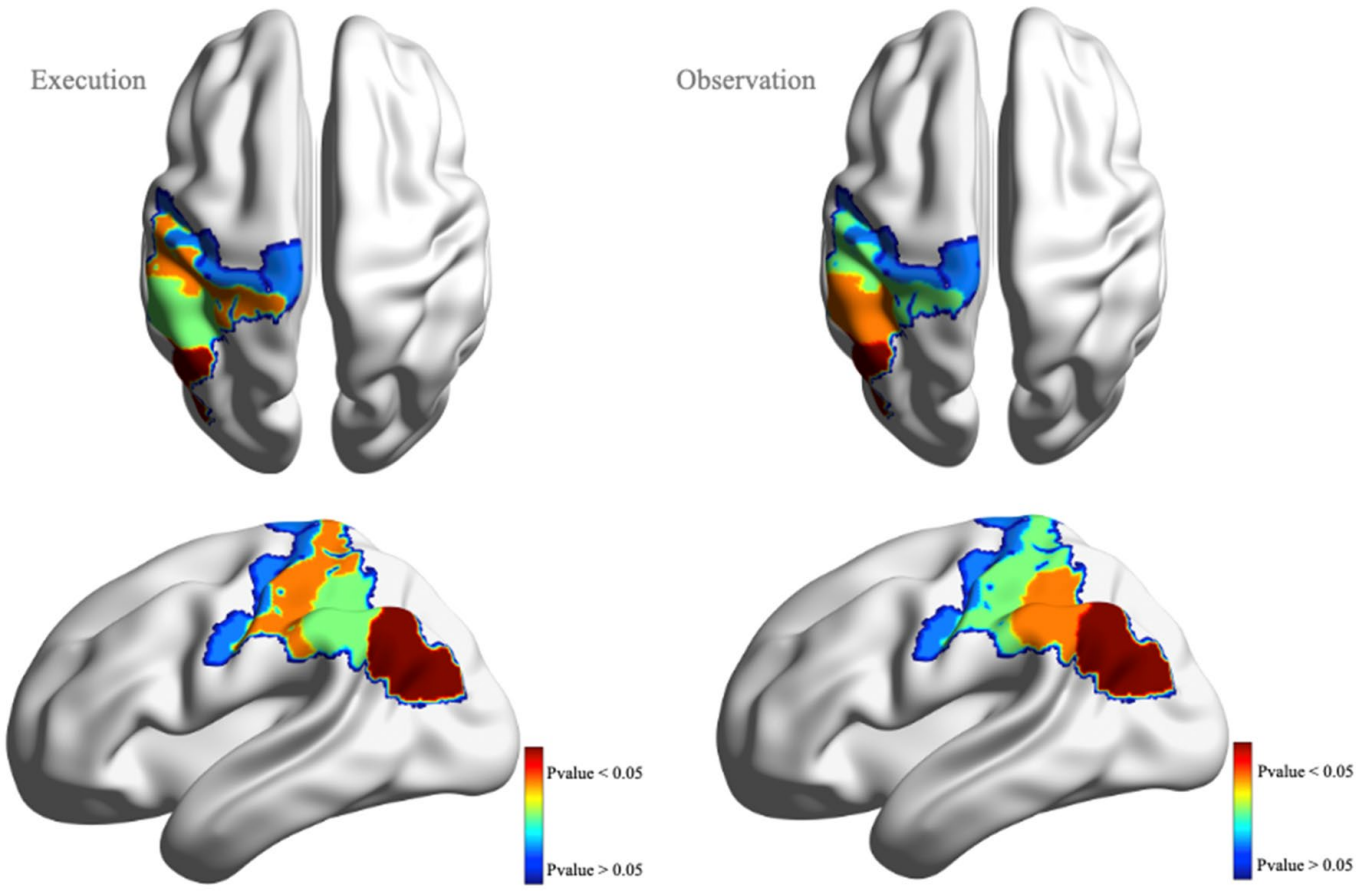
Extraced brain regions associated with execution (axial view on the top left, sagittal view on the bottom left) and observation (axial view on the top right, sagittal view on the bottom right). The color bar refers to the significance of the region (component). Our analysis shows the left inferior parietal lobule is the region which shows the highest covariation in fNIRS and EEG recordings (the most significant component). Interestiingly, the covariation in fNIRS and EEG signals in the right hemisphere are not shown to relate in our AON paradigm. Image generated using BrainNet Viewer software^63^.

We applied our proposed ssmCCA technique to analyze the correlation between EEG and fNIRS datasets of the 21 participants during the action execution and action observation conditions separately. Our ssmCCA aims to find canonical variates (components) that are the best representative of their own modalities and, at the same time, correlate robustly with the corresponding canonical variates of the other modality. Here, we extracted four canonical variates of which one was statistically significant. Table 1 shows these components, their corresponding correlation, and statistical significance for action execution and observation. Notably, the left parietal inferior region showed a significant correlation during action observation (*r* = 0.48, *p* = 0.041), and a marginally significant correlation during action execution (*r* = 0.39, *p* = 0.055). Figure 5 provides a schematic view of the brain regions associated with action execution and action observation.

**Table 1:** The brain regions and their cross modality correlation and corresponding Pvalues.

	Action execution				Action observation			
	Correlation	Corresponding fNIRS region	Corresponding EEG channel	P-value	Correlation	Corresponding fNIRS region	Corresponding EEG channel	P-value
Comp 1	0.3905	*Parietal_Inf_L*	E51, E52, E53, E59, E60	0.055	0.4823	*Parietal_Inf_L*	E52, E53, E59, E60	0.0416
Comp 2	0.3821	*Postcentral*	E37, E41, E42	0.0572	0.3753	*Supra-marginal-L*	E66, E69, E70, E71, E74	0.0512
Comp 3	0.3548	*Supra-marginal_L*	E66, E69, E70	0.0605	0.3310	*Postcentral L*	E37, E41, E42	0.0624
Comp 4	0.3350	*Precentral_L*	E19, E20, E24, E27	0.0647	0.3351	*Precentral_L*	E19, E20, E27	0.0762

**Figure 6:**
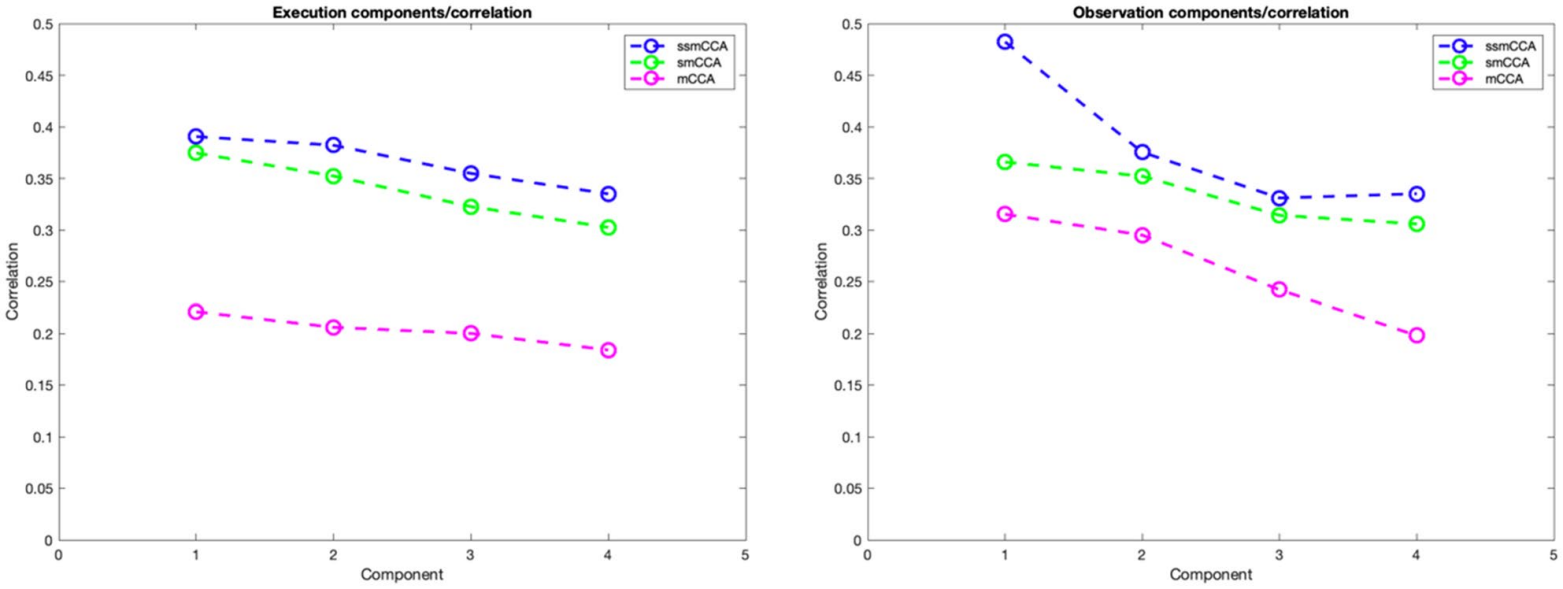
Correlations by their corresponding components resulting from EEG-fNIRS fusion applying mCCA, smCCA and ssmCCA.

Moreover, we examined the performance of our ssmCCA approach to two other common CCA approaches, smCCCA and mCCA. In Fig. 6, the correlations of components 1 to 4 are plotted across all three fusion approaches, with ssmCCA showing the largest correlation magnitudes across all four components in both the action and observation conditions.

## Discussion

Recent studies have shown great potential in combining multimodal brain imaging data captured from multiple participants by leveraging the rich information each modality provides. In this study, we developed a ssmCCA algorithm to explore relationships between multi-modal datasets to take advantage of the relative strengths of both EEG and fNIRS. The focus of this work was to use multimodal, multi-participant data fusion to characterize the human AON while addressing common problems in medical data processing, such as smaller sample size and other CCA methodological challenges, and show greater specificity in findings using a multimodal approach than the unimodal approaches alone. It is worth noting that the significance of this work is two-fold: 1. It provides methodological advancement in the field of multimodel imaging, and 2. It addresses an important research question affording better understanding of the AON.

Our proposed ssmCCA is an unsupervised learning algorithm which finds canonical variates without any prior information. As the quality and interpretibilty of the CCA components depend on the usefulness and relevance of each set of extracted features that are active across sets, our proposed ssmCCA model combines mCCA with *l1*-norm type penalty to automatically remove irrelevant features (sparsity constraint). This feature selection enhancement also mitigates overfitting problems caused by high-dimensional data sets with few correlated components and a small sample size. Adding *l2*-norm penalty, we assure the correlation between canonical projections is maximized without neglecting the local structure of the data (i.e., the adjacency in brain regions). Figure 6 also shows the advantage of using ssmCCA over smCCA (without considering *l2*-norm penalty) and mCCA (with no consideration of *l1*-norm and *l2*-norm penalties). As shown, applying those regularizations has improved the correlation between the two datasets and provided us with more accurate representation. Not only has our study contributed to the advancement of a method to combine EEG and fNIRS datasets, but also added to the AON literature by identifying a candidate ROI of the AON system in humans.

Our results are consistent with the AON literature. The ssmCCA analysis indicates canonical components in the inferior parietal, postcentral, supra-marginal, and precentral regions of the left hemisphere when participants performed action execution. As is well-established, motor execution engages mostly the contralateral sensory-motor cortex^64–67^, indexed by the precentral and postcentral regions in our study. Since participants in this study were all right-handed, the major components were detected on the left side of the brain. In the observation condition, we see the extracted components in inferior parietal, supra-marginal, postcentral, and precentral regions in the left hemisphere. Although the same sensory-motor regions are activated, the more dominant regions seem to be more posterior during the observation condition, as found in previous studies^11,68^. More importantly, the left inferior parietal lobe is shown to undergo the highest covariation (equivalent to greater oxyhemoglobin/decreased μ power) of simultaneous EEG and fNIRS data across both action execution and action observation, indicating that this is the strongest AON candidate region. Several unimodal studies have indicated involvement of many of these regions during action execution and action observation^15,69,70^. Our fNIRS unimodal analysis also implicated these regions amongst a larger set of areas of the brain involved in the human AON Fig. 3^53^. The unimodal fNIRS findings showed widespread activation across the parietal regions, including bilateral superior parietal lobule, bilateral inferior parietal lobule, right supra-marginal region and right angular gyrus, during action execution and action observation, whereas our multimodal analysis was able to identify regions with more specificity than the unimodal approach.

In addition, our results from the EEG unimodal analysis shows μ desynchronization in both execution and observation conditions across the cortex, with limited spatial specificity of μ desynchronization Fig. 4. However, we do see a “hemisphere effect” during execution such that there was greater activity in the left central compared to right central regions, which is consistent with the contralateral effect seen in our multimodal findings. However, there was no hemisphere effect in the observation condition when using the unimodal EEG analysis, thus characterization of the AON through EEG alone was not specific to one hemisphere. Therefore, the findings from our data fusion analysis appear to be consistent with both unimodal analyses while also more specific, pointing to the left inferior parietal lobe as the region that presents the highest covariation between EEG and fNIRS signals during an AON paradigm.

While the results from our study support the use of ssmCCA to fuse simultaneous EEG and fNIRS data in an attempt to better characterize the spatial profile of cortical activation during an AON paradigm, there were limitations that could be addressed in future research. For the sake of understanding the AON, especially given our findings of lateralized components, it will be important for future studies to include left-handed participants. Furthermore, we offered a comparison of the ssmCCA findings to the fNIRS alone and EEG alone findings; however, the EEG alone findings did not utilize source localization estimates. Given that sources for surface EEG are generally understood to originate from throughout the brain and show minimal spatial specificity, EEG source localization estimates would be a useful comparison to ssmCCA findings from the multimodal dataset, or even to use in the ssmCCA to further increase specificity of our findings. Lastly, the simultaneous collection of fNIRS and high density EEG data (128 + electrodes) was challenging. Not only was the initial integration of the two caps difficult, but data loss in one or both modalities led to a notably smaller sample size for the multimodal analyses. One potential solution is to determine whether high density EEG offers an advantage when using ssmCCA, or if a similar result could be obtained using a more sparse array of EEG electrodes. Determining this could minimize cap integration issues while offering the same quality of multimodal findings and will be further investigated. Similarly, in order to clarify the role of ssmCCA in multimodal analyses, it will also be useful to apply ssmCCA to multimodal datasets that are collected both simultaneously and sequentially. It is possible that sequential data collection can provide similar multimodal findings as simultaneous data collection when using ssmCCA, which again would minimize the integration issues mentioned above. Finally, we can also apply ssmCCA to investigate the temporal components of the AON by focusing on the timing aspects of EEG timeseries as opposed to the spatial aspects, as done in the present study. Since EEG captures cortical dynamics in milliseconds, and our paradigm recorded different action markers, such as starting the action and lifting the object, we could characterize how the AON is firing in relation to each of these actions, as well as the sequence AON activation over the course of the paradigm. This would help determine whether the sequence and timing of AON activation is the same during both action execution and action observation or if there is a time lag or difference in the activation sequence. However, this would require enough spatial resolution to delineate which cortical areas are firing at different times over the course of the paradigm, which is why the present study focused on improving spatial resolution with this approach. Nonetheless, ssmCCA could also potentially be used to calculate accurate timing of the AON response between conditions, which may afford additional insight into AON function.

## Data Availability

All raw *nirs* and *mmf* (EEG raw data) files that support the findings of this study along with the method implementation ssmCCA are available on Dash, a NIH Data and Specimen Hub https://dash.nichd.nih.gov/ Mirror Network in At-Risk Infants [Study ID: currently the data is being transferred. The ID will be generated upon a completion of data transfer].

## References

[1] Horwitz B, Poeppel D (2002). How can EEG/MEG and fMRI/PET data be combined?. Hum. Brain Mapp..

[2] Nunez PL, Silberstein RB (2000). On the relationship of synaptic activity to macroscopic measurements: Does co-registration of EEG with fMRI make sense?. Brain Topogr..

[3] Condy EE (2021). Characterizing the action-observation network through functional near-infrared spectroscopy: A review. Front. Hum. Neurosci..

[4] Dashtestani H (2018). The role of prefrontal cortex in a moral judgment task using functional near-infrared spectroscopy. Brain and behavior.

[5] Al-Shargie F (2017). Assessment of mental stress effects on prefrontal cortical activities using canonical correlation analysis: an fNIRS-EEG study. Biomed. Opt. Express.

[6] Al-Shargie F (2017). Stress assessment based on decision fusion of EEG and fNIRS signals. IEEE Access.

[7] Bunge, S. A. & Kahn, I. Cognition: An overview of neuroimaging techniques (2009).

[8] Pfurtscheller G, Da Silva FL (1999). Event-related EEG/MEG synchronization and desynchronization: basic principles. Clin. Neurophysiol..

[9] Meltzoff AN (2007). ‘Like me’: A foundation for social cognition. Dev. Sci..

[10] Kuhlman WN (1978). Functional topography of the human mu rhythm. Electroencephalogr. Clin. Neurophysiol..

[11] Fox NA (2016). Assessing human mirror activity with EEG mu rhythm: A meta-analysis. Psychol. Bull..

[12] Hobson HM, Bishop DV (2016). Mu suppression—A good measure of the human mirror neuron system?. Cortex.

[13] Hobson HM, Bishop DV (2017). The interpretation of mu suppression as an index of mirror neuron activity: Past, present and future. R. Soc. Open Sci..

[14] Caspers S (2010). ALE meta-analysis of action observation and imitation in the human brain. Neuroimage.

[15] Molenberghs P (2012). Brain regions with mirror properties: A meta-analysis of 125 human fMRI studies. Neurosci. Biobehav. Rev..

[16] Rizzolatti G (2005). The mirror neuron system and its function in humans. Anat. Embryol..

[17] Van Overwalle F, Baetens K (2009). Understanding others' actions and goals by mirror and mentalizing systems: A meta-analysis. Neuroimage.

[18] Filimon F (2007). Human cortical representations for reaching: Mirror neurons for execution, observation, and imagery. Neuroimage.

[19] Jelsone-Swain L (2015). Action processing and mirror neuron function in patients with amyotrophic lateral sclerosis: An fMRI study. PLoS ONE.

[20] Tulay EE (2019). Multimodal neuroimaging: basic concepts and classification of neuropsychiatric diseases. Clin. EEG Neurosci..

[21] Hotelling, H. CCA: An r package to extend canonical correlation analysis. Biometrika (1936).

[22] Kettenring. Canonical analysis of several sets of variables. Biometrika (1971).

[23] Dashtestani H (2019). Canonical correlation analysis of brain prefrontal activity measured by functional near infra-red spectroscopy (fNIRS) during a moral judgment task. Behav. Brain Res..

[24] Deleus F, Van Hulle MM (2011). Functional connectivity analysis of fMRI data based on regularized multiset canonical correlation analysis. J. Neurosci. Methods.

[25] Khalid, M. U. & Seghouane, A.-K. Multi-subject fMRI connectivity analysis using sparse dictionary learning and multiset canonical correlation analysis. 2015 IEEE 12th International Symposium on Biomedical Imaging (ISBI), IEEE (2015).

[26] Li Y-O (2009). Joint blind source separation by multiset canonical correlation analysis. IEEE Trans. Signal Process..

[27] Li YO (2012). Group study of simulated driving fMRI data by multiset canonical correlation analysis. J. Signal Process. Syst..

[28] Katthi, J. R. & Ganapathy, S. Deep Multiway Canonical Correlation Analysis for Multi-Subject EEG Normalization (2021). arXiv preprint arXiv:2103.06478.

[29] Zhang Y (2017). Sparse Bayesian multiway canonical correlation analysis for EEG pattern recognition. Neurocomputing.

[30] Correa NM (2010). Multi-set canonical correlation analysis for the fusion of concurrent single trial ERP and functional MRI. Neuroimage.

[31] de Cheveigné A (2019). Multiway canonical correlation analysis of brain data. Neuroimage.

[32] Adalı, T. *et al.* Special section on multimodal biomedical imaging: Algorithms and applications (2013).

[33] Correa NM (2008). Canonical correlation analysis for feature-based fusion of biomedical imaging modalities and its application to detection of associative networks in Schizophrenia. IEEE J. Sel. Top. Signal Process..

[34] Lahat D (2015). Multimodal data fusion: An overview of methods, challenges, and prospects. Proc. IEEE.

[35] Al-Shargie F (2016). Mental stress assessment using simultaneous measurement of EEG and fNIRS. Biomed. Opt. Express.

[36] Alyan E (2020). Investigating frontal neurovascular coupling in response to workplace design-related stress. IEEE Access.

[37] Witten DM (2009). A penalized matrix decomposition, with applications to sparse principal components and canonical correlation analysis. Biostatistics.

[38] Tibshirani R (2005). Sparsity and smoothness via the fused lasso. J. R. Stat. Soc. Ser. B (Stat. Methodol.).

[39] Witten, D. M. and R. J. Tibshirani (2009). "Extensions of sparse canonical correlation analysis with applications to genomic data." Stat Appl Genet Mol Biol **8**: Article28. 10.2202/1544-6115.1470 PMC2861323 19572827

[40] Hastie, T. *et al.* Statistical Learning with Sparsity: The Lasso and Generalizations, Chapman and Hall/CRC (2019).

[41] Rasmussen MA, Bro R (2012). A tutorial on the Lasso approach to sparse modeling. Chemom. Intell. Lab. Syst..

[42] Simon N (2013). A sparse-group lasso. J. Comput. Graph. Stat..

[43] Chen J (2013). Structure-constrained sparse canonical correlation analysis with an application to microbiome data analysis. Biostatistics.

[44] Du L (2015). GN-SCCA: GraphNet based sparse canonical correlation analysis for brain imaging genetics. Brain Inf. Health.

[45] Grosenick L (2013). Interpretable whole-brain prediction analysis with GraphNet. Neuroimage.

[46] Yang, S., et al. Feature Grouping and Selection Over an Undirected Graph. KDD: 922–930. 10.1145/2339530.2339675 PMC3763852 24014201

[47] He X (2004). Locality preserving projections. P. Niyogi. Neural Inf. Process. Syst.

[48] Mohammadi-Nejad, A.-R. Discovering true association between multimodal data sets using structured and sparse canonical correlation analysis: A simulation G.-A. Hossein-Zadeh, *IEEE 13th International Symposium on Biomedical Imaging (ISBI)* 820–823 (2016).

[49] Mohammadi-Nejad AR (2017). Structured and sparse canonical correlation analysis as a brain-wide multi-modal data fusion approach. IEEE Trans. Med. Imaging.

[50] Lin D (2014). Correspondence between fMRI and SNP data by group sparse canonical correlation analysis. Med. Image Anal..

[51] Waaijenborg, S., *et al.* Quantifying the association between gene expressions and DNA-markers by penalized canonical correlation analysis. *Stat. Appl. Genet Mol Biol.* **7**(1): Article3. 10.2202/1544-6115.1329 18241193

[52] Cannon EN (2016). Relations between infants' emerging reach-grasp competence and event-related desynchronization in EEG. Dev. Sci..

[53] Miguel HO (2021). Cerebral hemodynamic response during a live action-observation and action-execution task: A fNIRS study. PLoS ONE.

[54] Delorme A, Makeig S (2004). EEGLAB: An open source toolbox for analysis of single-trial EEG dynamics including independent component analysis. J. Neurosci. Methods.

[55] Debnath R (2020). The Maryland analysis of developmental EEG (MADE) pipeline. Psychophysiology.

[56] Nolan H (2010). FASTER: Fully automated statistical thresholding for EEG artifact rejection. J. Neurosci. Methods.

[57] Mognon A (2011). ADJUST: An automatic EEG artifact detector based on the joint use of spatial and temporal features. Psychophysiology.

[58] Cooper RJ (2012). A systematic comparison of motion artifact correction techniques for functional near-infrared spectroscopy. Front. Neurosci..

[59] Delpy DT (1988). Estimation of optical pathlength through tissue from direct time of flight measurement. Phys. Med. Biol..

[60] Koizumi H (2003). Optical topography: practical problems and new applications. Appl. Opt..

[61] Aasted CM (2015). Anatomical guidance for functional near-infrared spectroscopy: AtlasViewer tutorial. Neurophotonics.

[62] Lindquist MA (2009). Modeling the hemodynamic response function in fMRI: Efficiency, bias and mis-modeling. Neuroimage.

[63] Xia M (2013). BrainNet Viewer: A network visualization tool for human brain connectomics. PLoS ONE.

[64] Balconi M (2017). Transitive versus intransitive complex gesture representation: a comparison between execution, observation and imagination by fNIRS. Appl. Psychophysiol. Biofeedback.

[65] Balconi M (2015). Transitive and intransitive gesture execution and observation compared to resting state: The hemodynamic measures (fNIRS). Cogn. Process..

[66] Hardwick RM (2018). Neural correlates of action: Comparing meta-analyses of imagery, observation, and execution. Neurosci. Biobehav. Rev..

[67] Hardwick RM (2013). A quantitative meta-analysis and review of motor learning in the human brain. Neuroimage.

[68] Debnath R (2019). Mu rhythm desynchronization is specific to action execution and observation: Evidence from time-frequency and connectivity analysis. Neuroimage.

[69] Bhat AN (2017). Cortical activation during action observation, action execution, and interpersonal synchrony in adults: A functional near-infrared spectroscopy (fNIRS) study. Front. Hum. Neurosci..

[70] Crivelli D (2018). Linguistic and motor representations of everyday complex actions: an fNIRS investigation. Brain Struct. Funct..

